# Mitigating methane emissions: Domestic and joint efforts by the United States and China

**DOI:** 10.1016/j.ese.2024.100398

**Published:** 2024-02-06

**Authors:** Fan Dai, Yi Wang

**Affiliations:** a California-China Climate Institute, The University of California, Berkeley, USA; b Institutes of Science and Development, Chinese Academy of Sciences, Beijing, China; c University of Chinese Academy of Sciences, Beijing, China

During the Glasgow climate conference, the United States (U.S.) and China jointly committed to reduce methane emissions as a prioritized area of their bilateral cooperation in addressing climate change in the 2020s. Recognizing methane's strong warming effect, which is over 80 times more potent than carbon dioxide in the first 20 years after release, reducing methane emissions is crucial to slowing down climate change in the near future [1].^1^ The significance of this joint commitment was reiterated in the recent U.S.-China Sunnylands Statement on Climate Cooperation, emphasizing the urgency and importance of reducing methane emissions both within each country's borders and through collaborative efforts.

The U.S. and China are the third and first largest methane emitters globally, accounting for 31.2 and 52.8 million tons, respectively, in 2023 [2]. While the U.S. has initiated the Energy Pathway of the Global Methane Pledge^2^ to tackle routine flaring in fossil fuel operations and cut methane pollution in the oil and gas sector, it still needs to make more efforts to reduce methane from the agricultural and waste sectors. Conversely, China's methane plan, unveiled in November 2023, outlines strategies for methane reduction across the energy, agriculture, and waste sectors [3]. Notably, this plan lacks specific, measurable targets. The Global Methane Pledge was deemed too ambitious for China, which, as an upper middle-income country, may face challenges in acquiring the necessary data, monitoring technology, and effective measures to fully control non-CO_2_ greenhouse gases (GHGs) [4].

Despite escalating bilateral tensions and political differences, the U.S. and China share a common goal of reducing methane emissions and mitigating the effects of climate change. Both countries face similar challenges in implementing effective policies and actions to achieve this goal. This paper aims to assess the potential for reducing methane emissions in key sectors, the governance and policy challenges at the national and sub-national levels, and identify opportunities for collaboration. By exploring these three areas, the paper offers solutions and prospects for U.S.-China cooperation in reducing methane emissions.

## 1 Abatement potential in key sectors

In the U.S., the energy and agriculture sectors are the primary sources of methane emissions. The oil and gas industry and coal mining are responsible for the majority of energy-related methane emissions, contributing a total of 40% of the nation's methane emissions. Meanwhile, the agriculture sector accounts for 45% of methane emissions, mainly from enteric fermentation. In China, the total methane emissions in 2014 were 55.29 million tons (1161 million tons of CO_2_ equivalent (CO_2_e), 100-year Global Warming Potential), accounting for 10.4% of national GHG emissions [5]. The energy sector, predominantly influenced by coal mining, generates 44.8% of methane emissions, while agriculture accounts for another 40.2%.

**Fig. 1:**
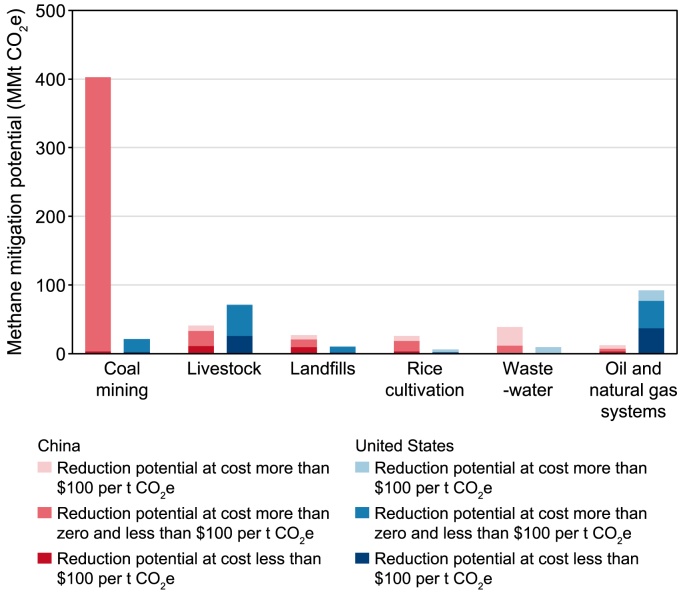
Methane mitigation potential by cost in key sectors in the U.S. and China [8].

**Fig. 2:**
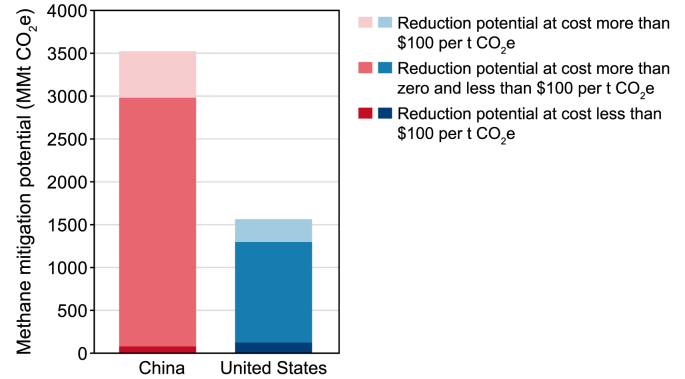
Methane mitigation potential by cost [8].

The energy supply (coal, oil, and gas), agriculture, and waste sectors are the major sources of methane emissions. Currently, available measures have the potential to reduce global methane emissions from these sectors by 45% by 2030 compared to projected emissions [6]. Both the U.S. and China have significant potential to reduce their methane emissions in the next decade, including low-cost reductions, as shown in Fig. 1, Fig. 2. The U.S. has a total methane reduction potential of 224 million metric tons of CO_2_ equivalent (Mt CO_2_e) in 2030 using abatement measures at or below $100 per metric ton of CO_2_e [7]. In China, the methane reduction potential is estimated to reach 469 Mt CO_2_e in 2030, equivalent to a 35% reduction from 2015 levels [9]. The projected upper limits for methane reduction by 2030, compared to a Business-as-Usual scenario, display a noteworthy similarity, with estimates ranging from 38% to 41% [10,11].

### 1.1 Energy supply

Globally, the energy sector (coal, oil & gas) holds significant potential for reducing methane emissions, which were found to be 70% higher than officially reported, according to IEA. The primary reduction potential lies in the oil and gas sector, where the global reduction potential is 29–57 million metric tons of methane (Mt CH_4_) per year [6]. The U.S. and China both have significant mitigation potential in their energy sectors, with the greatest reduction potential coming from the oil and gas sector. The U.S. has a mitigation potential of 144 Mt CO_2_e up to $100 per metric ton of CO_2_e, while China's energy sector (including coal mining and oil & gas) has a reduction potential of 308–407 Mt CO_2_e with abatement costs below/up to $100 per metric ton of CO_2_e [9,10]. Reducing methane emissions from coal mining can also provide potential reductions of 12–25 Mt CH_4_ per year [6]. China has a higher reduction potential in coal mining, with 256–399 Mt CO_2_e of reduction potential in 2030 [9,10]. Reducing methane emissions from these sectors can be achieved through measures such as ventilation air methane oxidation, recovery and use of methane, upgrades to equipment, changes in operational practices, monitoring measures, and management of abandoned coal mines.

### 1.2 Agriculture

Globally, improvements in feeding and manure management within the livestock sub-sector are projected to reduce 4–42 Mt CH_4_ per year in the agriculture sector. Additionally, enhanced water management and improved yield gains in rice cultivation are anticipated to contribute to a reduction of 6–9 Mt CH_4_ per year [6]. In the U.S., the agriculture sector's methane reduction potential is estimated at 72 Mt CO_2_e (at $100 per metric ton of CO_2_e), with most coming from the livestock sub-sector. In China, the agriculture sector's reduction potential is estimated at 65–116 Mt CO_2_e for similar abatement costs.

### 1.3 Waste

In the waste sector, global reduction potential is estimated at 29–36 Mt CH_4_ per year [6]. The U.S. and China have similar shares of methane emissions from landfills and waste and mitigation measures include controlling and flaring emissions, utilizing landfill gas, and reducing organic waste in landfills and solid waste. The U.S. has a mitigation potential of 8 Mt CO_2_e in 2030 at an abatement cost of $100 per metric ton of CO_2_e [7], while China's potential is 26–45 Mt CO_2_e in 2030 [9,10].

## 2 Governance and policy challenges at the national and sub-national levels

To address the climate crisis and reduce methane emissions, it is crucial to implement policies at both national and subnational levels. The U.S. has committed to a 50–52% reduction in net GHG emissions below 2005 levels by 2030, as stated in their nationally determined contribution (NDC) target ahead of COP 26 [12]. The U.S. has also released a Methane Emissions Reduction Action Plan to reduce methane emissions from oil and gas infrastructure, landfills, and agriculture. In California, for example, the current policy requires a 40% reduction of methane emissions by 2030 compared to 2013 levels [13]. These actions show the need for comprehensive efforts to tackle the problem of methane emissions and mitigate the impact of climate change.

China has incorporated the imperative to effectively control and enhance the management of GHG emissions, including methane, in its 12th, 13th, and 14th Five-Year Plans. In 2021, China's recently announced 2060 carbon neutrality pledge includes non-CO_2_ GHGs such as methane [14],^3^ and its updated NDCs also include actions to address methane emissions. In November 2023, China released the Methane Emission Control Action Plan, which set qualitative methane control targets for the 14th and 15th Five-Year Plan periods. This comprehensive plan delineates specific measures for methane inventory establishment and mitigation in the energy, agriculture, and waste sectors. Notably, it incorporates overarching strategies to concurrently control methane and other pollutants, promote technological innovation and methane monitoring, and refine technical standards for various methane sources.

Despite the progress made in the U.S. and China, there are several major challenges in the governance structure and policy of methane emission reduction.

### 2.1 Political uncertainty and lack of holistic approach

Challenges in methane emission reduction include inconsistent policies and uncertain political leadership. In the U.S., the outcome of the 2022 elections can impact further action on methane emissions, with the possibility of regulatory rollbacks by the next White House administration. Additionally, the Environmental Protection Agency (EPA)'s authority to regulate the electricity sector faces legal scrutiny, which may pose hurdles for forthcoming regulations. Moreover, the absence of comprehensive plans in certain subsectors exacerbates these challenges. Both the U.S. and China are in the nascent stages of developing technological strategies for methane mitigation. This hampers the implementation of effective reduction efforts at both the national and sub-national levels.

In the realm of methane emission reduction, practical challenges persist despite political leadership and commitment. The U.S. has limited strategies and technology for mitigating non-CO_2_ GHGs in some subsectors. Meanwhile, China lacks comprehensive regulations and frameworks for managing methane emissions. Although China recently released a long-awaited plan to tackle methane, the language in the plan is relatively general. The plan lacks a firm, quantitative methane mitigation target, and most of its quantitative goals are not new but rather extensions of existing targets in the 14th Five-Year Plans. To mitigate methane, China still needs to establish a comprehensive policy framework, including an ambitious methane reduction target and specific working plans for the sectors outlined in the national action plan. In addition, in terms of geopolitical tension and energy supply guarantee responsibility in recent years, the potential of methane reduction would be influenced in some degree.

### 2.2 Data transparency and integrated inventory-monitoring-reporting-verification system

The sources of non-CO_2_ emissions are diverse, requiring specific strategies for each subsector and gas. Although significant progress has been made in tracking methane emissions using advanced technology, significant gaps still remain. In the U.S., a key step forward involves enhancing data transparency and adopting a more integrated approach to inventory and monitoring. China currently lacks a comprehensive monitoring and reporting system for non-CO_2_ GHGs, including methane, with limited data on baseline emissions and outdated information from 2014. Existing policies and measures are predominantly qualitative, lacking systematic tracking and evaluation. For example, the 2008 Standard on Coal Mine Methane Emissions in China has also not been effectively tracked or monitored, making its effectiveness unclear. This highlights the need for better data collection and tracking of methane emissions in both the U.S. and China.

### 2.3 Federal-state coordination

Effective reduction of methane emissions requires federal-state coordination. In the U.S., policymaking is divided between federal and sub-national governments, with Congress and the Executive Branch holding federal authority and states, counties, and cities holding sub-national authority. The EPA leads the federal Methane Action Plan, while sub-national governments regulate specific areas such as agriculture, landfills, and gas pipelines. In California, SB 1383 authorizes state agencies to regulate methane from livestock, waste, and landfills. In China, policymaking is mostly led by the national government, but details on implementation in major emitting sectors are still unclear. Effective action requires coordination between national and sub-national governments.

Moreover, inadequate support from science, research, and technology could impede the formulation and implementation of methane policies. In the U.S. and European Union, extensive techno-economic analysis on methane has been conducted, whereas in China, there is limited cost data for mitigation measures and technologies, except for rice cultivation. The absence of cost analysis data makes policy-making and action slower as relevant information is lacking. More financial resources are required to support methane reduction policies and programs.

## 3 Opportunities for collaboration

Given the pressing nature of the climate crisis and the large discrepancy between current efforts and the targets outlined in the Paris Agreement, the U.S. and China have committed to taking individual and joint actions, coupled with international cooperation, in this crucial decade. Through these efforts, the two nations can demonstrate globally how swift action and effective collaboration can be achieved. During the UNFCCC COP 28, U.S., China and the UAE co-hosted a methane summit, which demonstrated cooperation opportunities on technical solutions, policy and capcity building.

### 3.1 A clear path of policy prioritization

To effectively reduce methane emissions, both China and the U.S. need to adopt a clear and decisive approach. This approach should include clear quantitative targets and timelines, prioritized mitigation strategies in key sectors, and a range of policy tools to support implementation. Both nations should aim to exceed their current climate targets and consider incorporating quantifiable reduction targets in their national climate strategies based on capacity and feasibility, respectively. Key sectors such as energy, agriculture, and waste must be prioritized. The U.S. should promptly enact regulations for reducing oil and gas emissions and partner with states to achieve a 70% reduction in methane emissions from large landfills. China, on the other hand, should embrace emerging international protocols to prevent leakage in the energy sector. To further enhance the comprehensive approach, market-based incentives and voluntary programs should complement the regulations and mandates. Moreover, the U.S. and China can cooperate in areas such as plugging abandoned gas and oil wells, promoting heat pump technology, and assisting disadvantaged communities to ensure a socially responsible transition to clean energy.

### 3.2 Facility-level measurement, reporting, and verification (MRV) and inventory

To effectively reduce methane emissions, a comprehensive set of tools must be in place. Among these tools, creating methane emissions inventories and establishing a baseline stand out as crucial foundations for setting future targets. In addition, a facility-level measurement, reporting, and verification (MRV) system must be developed to accurately track reductions. Improving the accuracy of methane emissions data reporting and tracking is critical for ensuring a strong MRV system. This accuracy is instrumental for national and sub-national programs to meet their goals. The U.S. and China must take action to enhance their inventory and MRV systems to achieve meaningful reductions in methane emissions and see timely results.

### 3.3 Efficient institutional coordination

In order to achieve meaningful and effective results in reducing methane emissions, it is critical to have coordinated action at various levels, including multilateral, national, and sub-national. This requires establishing robust institutional frameworks to share cost-effective mitigation strategies. Furthermore, there is a need to enhance the understanding of methane mitigation's significance among various stakeholders, including government entities, media outlets, academia, industrial sectors, and the public.

### 3.4 Accelerated technological innovation and financial support

Establishing financial incentives, fostering public-private partnerships, and investing in research and development of new technologies are essential for supporting the implementation of mitigation measures. Besides of basic responsibility ruled by UNFCCC and Paris Agreement, the U.S. and China can collaborate to mobilize international financing, particularly in developing countries, to promote sustainable and low-carbon technologies and practices. This includes utilizing carbon pricing mechanisms and international funding sources, such as the Multilateral Development Banks, to support the transition to low-carbon economies, particularly in regions where technical and financial assistance is needed. Additionally, there is a need to incentivize the private sector to invest in developing new and innovative mitigation technologies to reduce methane emissions. The U.S. and China can collaborate to create a supportive regulatory environment for investment in new technologies and share information on best practices and lessons learned from their experiences.

### 3.5 Sub-national and local cooperation methane mitigation

Subnational entities, encompassing states, provinces, and cities, can play a pivotal role in implementing measures and deploying technological solutions and policies to mitigate methane emissions. Collaborative efforts at the subnational level between the United States and China have the potential to yield tangible policy outcomes. For instance, Governor Gavin Newsom's recent visit to China sparked discussions on pilot projects, including initiatives to reduce emissions from rice cultivation in Shanghai, implement a landfill methane reduction project in the same city, and undertake similar projects in California [15]. Furthermore, initiatives focusing on methane emission detection and monitoring, as well as the capture and utilization of methane in industrial parks, offer promising avenues for expediting reductions in methane emissions from industrial processes.

China may explore incorporating offset options for methane emission reductions within its national emissions trading scheme based on domestic and international experiences. Collaborative opportunities at the sub-national level exist for reducing methane emissions in the waste and agriculture sectors internationally. This could involve implementing policies and incentives to encourage the adoption of mitigation measures, such as oil and gas protocols in the U.S. states of New Mexico, Colorado, and California and agricultural methane reduction initiatives in California.

## Footnotes

1 Strong evidence suggests that the increase in the atmospheric abundance of methane is due to increased emissions from fossil fuel activities, agriculture, and waste sources. Taking targeted action on reducing methane could avoid nearly 0.3 Celsius of global warming by the 2040s [6], thus providing more time to adopt more ambitious action to reduce carbon emissions.

2 The Global Methane Pledge which was first announced at COP26 in Glasgow in November 2021, now has been joined and endorsed by 120 countries, representing about three quarters of the global economy.

## Footnotes

3 https://news.bjx.com.cn/html/20210726/1165861.shtml.
